# Microembolic signal detection in acute ischemic stroke: Clinical relevance and impact on treatment individualization—A narrative review

**DOI:** 10.1111/ene.16584

**Published:** 2024-12-20

**Authors:** Eleni Bakola, Lina Palaiodimou, Odysseas Kargiotis, Apostolos Safouris, Klearchos Psychogios, Theodoros Karapanayiotides, Christos Moschovos, Vijay K. Sharma, Mark N. Rubin, João Sargento Freitas, Claudio Baracchini, Christos Krogias, Andrei V. Alexandrov, Tsivgoulis Georgios

**Affiliations:** ^1^ Second Department of Neurology School of Medicine, “Attikon” University Hospital, National and Kapodistrian University of Athens Athens Greece; ^2^ Stroke Unit Metropolitan Hospital Piraeus Greece; ^3^ Second Department of Neurology School of Medicine, Aristotle University of Thessaloniki Thessaloniki Greece; ^4^ Division of Neurology YLL School of Medicine, National University of Singapore, National University Hospital Singapore Singapore; ^5^ Edward Hines Jr. Veterans Affairs Medical Center Hines Illinois USA; ^6^ Department of Neurology Universidade de Coimbra Faculdade de Medicina Coimbra Portugal; ^7^ Stroke Center and Neurosonology Laboratory, Department of Neuroscience Padua University Hospital Padova Italy; ^8^ Department of Neurology EvK Herne Herne Germany; ^9^ Department of Neurology Banner University Hospital, University of Arizona College of Medicine Phoenix USA

**Keywords:** acute cerebral ischemia, acute reperfusion treatment, ischemic stroke, microembolic signals, secondary stroke prevention, stroke subtypes, transcranial doppler

## Abstract

**Background:**

Microembolic signals (MES) can be detected using transcranial Doppler (TCD) ultrasound in several clinical scenarios, including acute ischemic stroke (AIS). This narrative review aims to provide insights into their role in AIS patient management and outcomes.

**Methods:**

The present narrative review consolidates current observational and randomized evidence on the prevalence and clinical relevance of MES in different AIS subtypes and settings.

**Results:**

MES prevalence is higher in AIS patients with large artery atherosclerosis, indicating unstable or vulnerable plaques, and lower in those with small vessel disease. Detecting MES can significantly aid in managing AIS patients, particularly when the cause is unclear, as MES detected in different cerebral arteries can indicate conditions like covert cardioembolism, aortic arch atherosclerosis, or coagulation disorders, including cancer‐related stroke. MES are associated with higher risk of stroke recurrence, independently of the underlying stroke mechanism. The detection of MES during and after acute systemic or endovascular reperfusion procedures in large‐vessel occlusion patients appears to be predictive of adverse clinical outcomes and recurrent stroke. Finally, a reduction in MES detection may serve as surrogate marker and intermediate endpoint evaluating secondary stroke prevention treatments in the settings of randomized‐controlled clinical trials.

**Conclusion:**

MES detection on TCD in AIS remains a useful diagnostic tool as it helps the clinicians to approach the stroke underlying mechanism by detecting and quantifying ongoing cerebral embolization and localizing an embolic source in real time. In addition, it allows monitoring and treatment individualization in stroke patients, while further determining recurrent stroke risk.

## INTRODUCTION

Over 30 years ago, it was recognized that it was possible to detect microemboli moving through the brain arteries with transcranial Doppler (TCD) ultrasound by recording high‐intensity transient signals [1]. Microembolic signals (MES) are characterized by short lasting (<0.01–0.03 s), unidirectional intensity increases within the Doppler spectrum that occur randomly in the cardiac cycle and produce a “clicking” sound as they pass through the sample volume. MES can be either gaseous or solid. Solid emboli are platelet‐rich aggregates, atheromatous material or fat, and gaseous emboli are gaseous microbubbles, for example, due to cavitation at mechanical heart valves. Emboli made of different materials return signals of varying intensities. For instance, air has an acoustic impedance less than 1/4000th of that of blood, leading to a very strong reflection. Conversely, solid emboli have an acoustic impedance closer to that of blood, resulting in a much weaker reflection. In other words, bubbles reflect ultrasound more efficiently than solid materials, causing higher mean intensity increases. However, with conventional TCD equipment it is impossible to discriminate between gaseous and solid emboli or between platelet and atheroma emboli. Notably, administering 100% oxygen during TCD‐monitoring in patients with mechanical heart valves has been shown to reduce the occurrence of MES compared to room air. This reduction, along with the decreased intensity of signals under oxygen, may provide a useful approach to differentiating between gaseous and non‐gaseous emboli [2].

Detecting MES in stroke patients holds significant clinical importance, offering insights into the mechanisms of stroke and guiding targeted interventions. The aim of the present narrative review is to summarize current observational and randomized evidence evaluating the prevalence and the clinical significance of MES in different settings of acute ischemic stroke (AIS).

## METHODS

We performed a detailed search in MEDLINE, SCOPUS, Cochrane Library, and Google scholar up to November 1, 2024, using the following terms in combination: (microembolic signals OR microemboli OR MES) AND (stroke OR cerebral infarction OR cerebral ischemia OR TIA) AND (transcranial doppler sonography OR TCD OR transcranial ultrasonography). We included case–control, population‐based, and cohort studies that examined MES in patients with cerebrovascular ischemic events.

## PREVALENCE OF MES IN AIS AND DIFFERENT STROKE SUBTYPES

A cohort study, which aimed to determine the prevalence of MES and the potential diagnostic benefit in different stroke types according to TOAST classification, found an overall prevalence of 5.7% among 653 patients with AIS and transient ischemic attack (TIA) [3]. MES were significantly more common in patients with large artery atherosclerosis compared with other subtype groups. Another recent meta‐analysis confirmed that the prevalence of MES was high among all stroke subtypes except in patients with small vessel disease [4]. An overview of the presence of MES in different stroke etiologies is presented in Table 1.

**TABLE 1:** Frequency and arterial territories of the detection of microembolic signals among patients with ischemic stroke according to the stroke subtype or underlying stroke etiology.

Stroke subtype	Frequency of MES detection	Arterial territories of MES detection
Large artery atherosclerosis	+++ (30%)	Single
Carotid artery atherosclerosis	+++ (35%–45%)	Single
Intracranial atherosclerosis	+++ (15%–39%)	Single
Cardioembolic	++ (24%)	Multiple
Atrial fibrillation	++ (23%)	Multiple
Mechanical prosthetic valves	+++ (50%)	Multiple
Infective endocarditis	+++ (16%–40%)	Multiple
Small vessel disease	+ (3%–6%)	Single
Cryptogenic	++ (22%)	Single and multiple
ESUS	+++ (50%)	Single and multiple
Other determined etiologies	+++ (35%)	Single and multiple
Cancer‐related	+++ (32%–46%)	Multiple
Cervical artery dissection	+++ (50%)	Single
Aortic arch atheroma	+++ (13%–48%)	Multiple
Ascending aortic dissection	++ (25%)	Multiple
CNS Angiitis	++ (9%–15%)	Multiple
Moyamoya disease	+++ (12%–32%)	Multiple

*Note*: +/++/+++: low/moderate/high relevant frequency.

Abbreviations: CNS, central nervous system; ESUS, embolic stroke of undetermined source; MES, microembolic signals.

### Large artery atherosclerosis‐associated AIS

#### Carotid artery stenosis

**FIGURE 1:**
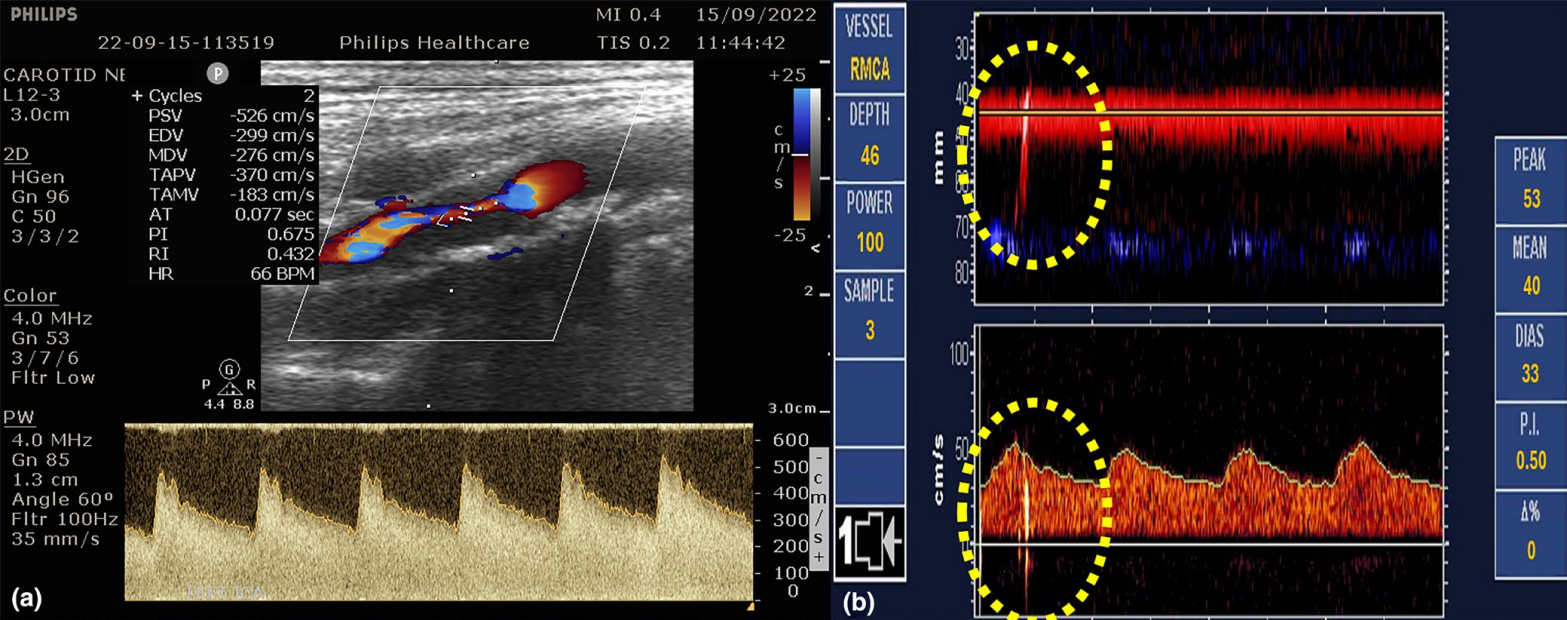
Patient with acute ischemic stroke due to symptomatic internal carotid artery stenosis, showing a hypoechoic plaque with a peak systolic velocity of more than 400 cm/sec causing >90% stenosis of the right internal carotid aartery (a) with positive microembolic detection on the ipsilateral middle cerebral artery as detected by Transcranial Power Motion‐Mode Doppler ((b) upper image) and in Doppler spectrum ((b) lower image).

**FIGURE 2:**
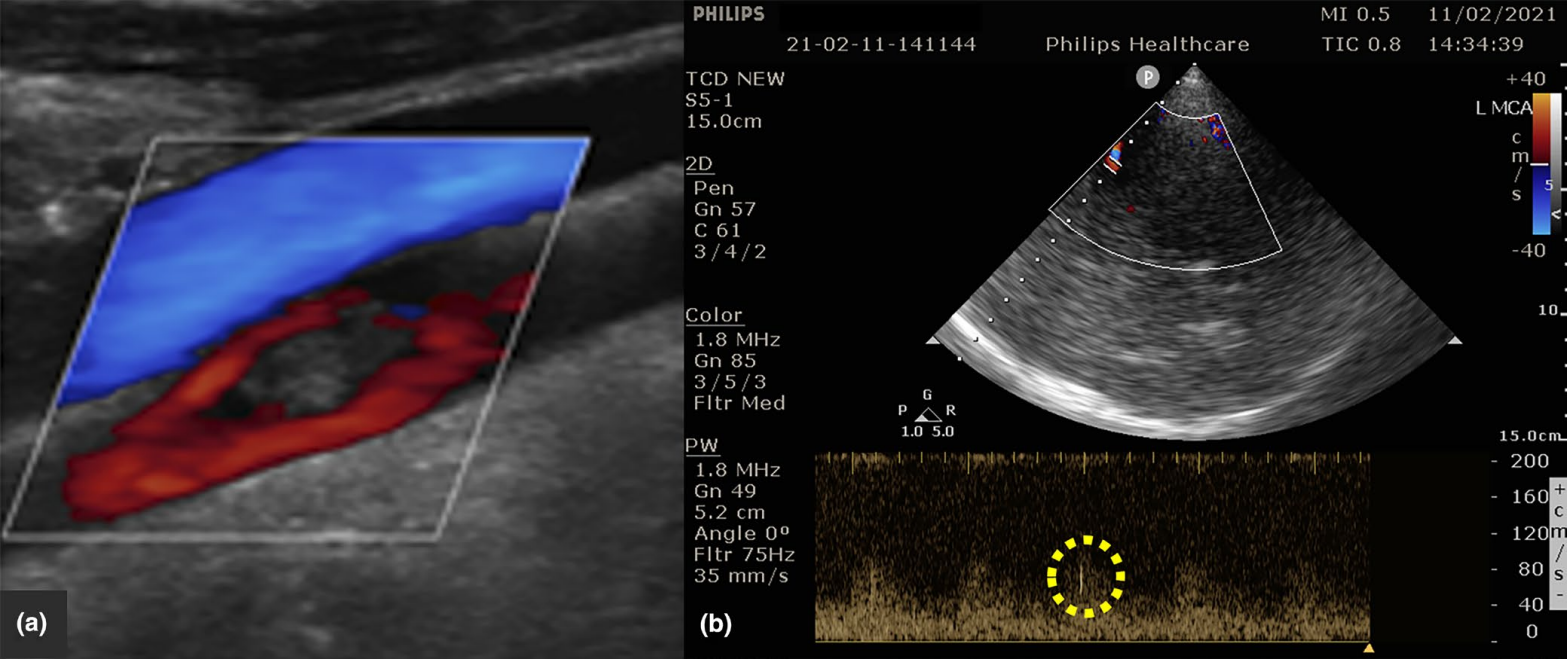
Patient with acute ischemic stroke due to floating thrombus in the left internal carotid artery (a) with positive microembolic detection on the ipsilateral middle cerebral artery as detected by Transcranial Color‐Coded Doppler (b).

**FIGURE 3:**
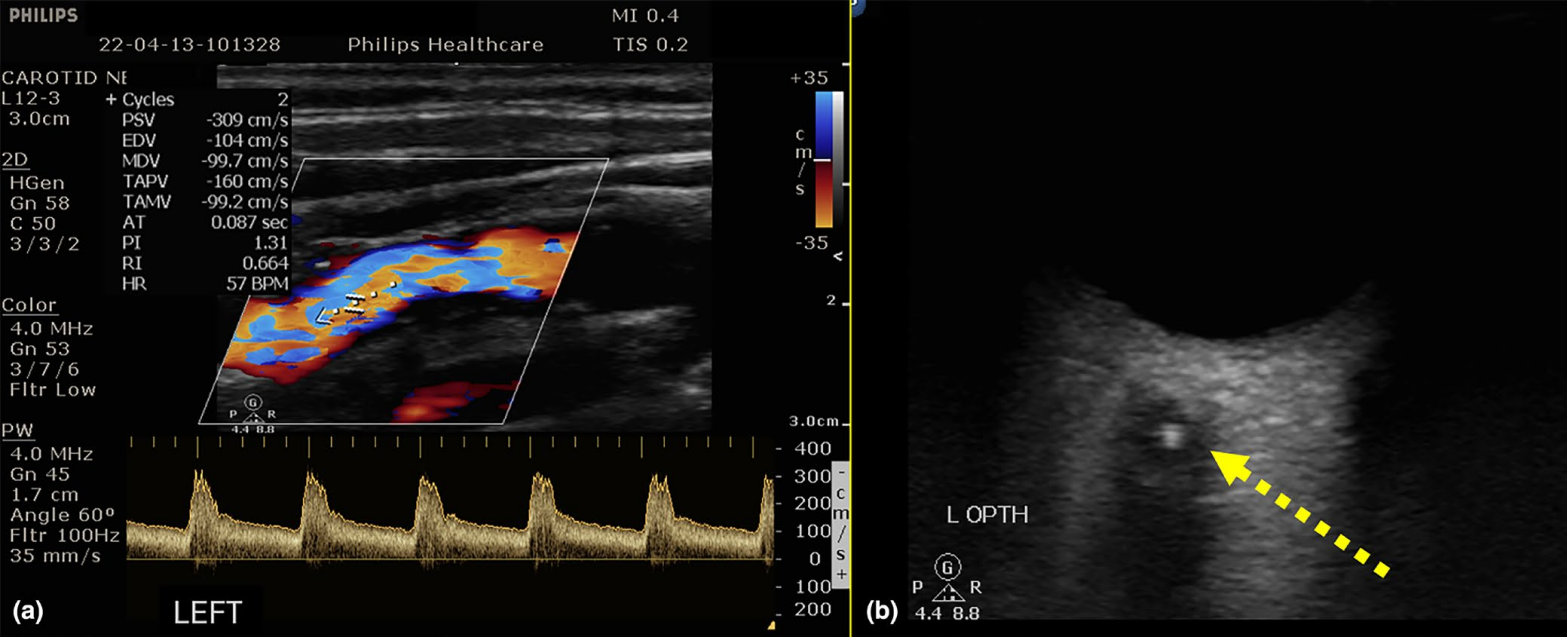
Patient with amaurosis fugax due to symptomatic internal carotid artery stenosis, showing a hypoechoic plaque with a peak systolic velocity of 309 cm/sec causing 70%–90% stenosis of the left internal carotid artery (a) with hyperechogenic retrobulbar spot sign in the central retinal artery as detected by Transorbital Sonography (b).

Numerous studies have identified MES in patients with symptomatic carotid stenosis (Figure 1). The presence of MES is more likely in patients who recently experienced stroke‐symptoms and patients with ulcerated plaques, intra‐plaque hemorrhages or floating carotid thrombi (Figure 2). Moreover, MES are more likely to be detected in patients with a higher degree of stenosis rather than in moderate stenosis [5]. In addition, unstable carotid plaques may also cause central retinal artery embolism that can be readily identified using transorbital sonography (retrobulbar spot sign; Figure 3).

MES have also been evaluated as a surrogate marker of effective secondary stroke prevention among patients with symptomatic carotid stenosis. The Clopidogrel and Aspirin for Reduction of Emboli in Symptomatic Carotid Stenosis (CARESS) trial was a multicenter randomized‐controlled clinical study, showing that clopidogrel‐load (300 mg) followed by dual antiplatelet therapy reduces asymptomatic embolization compared to monotherapy with aspirin 75 mg in patients with symptomatic carotid artery stenosis with positive MES detection [6]. In the intention‐to‐treat analysis there was a significant reduction of MES detection on TCD recordings in the dual‐therapy patients compared to the monotherapy group (43.8% vs. 72.7%, respectively). Furthermore, Tsivgoulis et al. highlighted the efficacy of clopidogrel‐load followed by dual antiplatelet treatment in significantly reducing the number of MES in symptomatic carotid artery stenosis prior to urgent carotid endarterectomy (CEA) without increasing the bleeding events [7].

Regarding the interventional management of carotid artery stenosis, MES detection on TCD could serve as a marker to expedite therapeutic interventions. Markus et al. reported on 200 patients with >50% symptomatic carotid stenosis; among them 89 presented MES on TCD in a 1‐h unilateral recording [8]. The presence of MES predicted stroke recurrence, while dual antiplatelet treatment and treating an asymptomatic stenosis (rather than a symptomatic one) were shown to be negatively associated with MES detection. Importantly, MES were more frequently detected after CAS compared to CEA [9].

Furthermore, the efficacy of statin pretreatment regarding MES detection, specifically due to atherosclerosis‐related AIS, was investigated in several studies, that were synthesized in a recent systematic review and meta‐analysis [10]. This study showed that statin pretreatment was significantly correlated with a reduced risk of MES detection during TCD‐monitoring.

More specifically, the detection of MES on TCD in AIS patients with large artery atherosclerosis may guide therapeutic interventions by increasing the duration of dual antiplatelet therapy (from 21 to 90 days) and the intensity of lipid‐lowering therapy.

#### Intracranial artery stenosis

The prevalence of MES in patients with intracranial stenosis of atherosclerotic origin has been investigated in several studies in the past decades in the context of AIS, but also in asymptomatic patients. The frequency of MES detection on TCD in patients with symptomatic intracranial stenosis ranges between 15% and 39% among the studies [11, 12]. On the other hand, MES were significantly less frequent among patients with asymptomatic compared to symptomatic intracranial stenosis or even absent [13].

Whether or not MES could serve as surrogate marker to predict stroke recurrence is still a debatable issue. Chen et al. reported that the risk of recurrent stroke was higher in patients in whom TCD was positive for MES compared with patients without MES on TCD‐monitoring, especially within the first 12 months [13]. These findings were in line with the study by Gao et al. that identified the presence of MES on TCD—in a 30 min recording—as the only predictor of recurrent stroke in AIS patients with middle cerebral artery (MCA) stenosis [14]. In contrast, Tan et al. found no relationship between MES and early stroke recurrence [11]. Similarly, the Mechanisms of earlY Recurrence in Intracranial Atherosclerotic Disease (MYRIAD) study also found no significant correlation between the presence of MES and stroke recurrence [12].

Moreover, the presence of MES on TCD in patients with intracranial stenosis and AIS raises issues regarding secondary prevention. The clopidogrel plus aspirin for infarction reduction in acute stroke or transient ischaemic attack patients with large artery stenosis and microembolic signals (CLAIR) trial was a randomized‐controlled clinical trial with the aim to evaluate the efficacy of short‐term dual antiplatelet treatment versus aspirin alone in reducing MES detection at 7‐day follow‐up among AIS patients with large artery atherosclerosis that additionally presented positive MES detection at baseline. After enrolling 100 patients, it was shown that indeed MES, as detected during a 30‐min recording, were significantly reduced among patients receiving dual antiplatelet treatment [15]. In a further subgroup analysis of CLAIR study that exclusively focused on patients with intracranial stenosis, Wang et al. found that in patients with MES treated with dual antiplatelet therapy, TCD demonstrated significantly lower number of MES on Day 2 after stroke onset compared to MES positive patients treated with single antiplatelet therapy [16].

#### Cardioembolic AIS

Atrial fibrillation (AF) is one of the main causes of cardioembolic AIS. As part of stroke prevention measures, AF should be promptly recognized and treated by effective anticoagulation. Apart from detecting rhythm abnormalities during neurosonological evaluation, the detection of MES in different vascular territories may indicate the presence of AF and an increased risk of cerebral embolism [17]. Furthermore, the presence of MES shortly after AIS attributed to newly diagnosed AF has been associated with worse functional outcomes [18]. MES detection may also reflect the effectiveness of anticoagulation in AF. In a previous study, a negative correlation was shown between MES presence and INR values in AF patients receiving warfarin: the higher INR values, the lower number of MES detected [19]. Rhythm control has recently gained attention in terms of stroke prevention, with AF ablation being a viable option. However, it is not uncommon for MES to be detected in a clustered, yet single, shower‐like pattern during ablation techniques. Thankfully, the detection of MES during ablation has not been associated with adverse neurological outcomes, either AIS or neuropsychological decline [20].

MES are frequently detected in valvular diseases and especially among patients with mechanical prosthetic valves. Furthermore, surgical treatment of valvular disease, that is, valve replacement, has also been associated with increased MES detection, and the presence of MES itself may be a predictor of postoperative neuropsychological deficits [21].

Another aspect for the usefulness of MES detection in clinical practice is in patients with suspected infective endocarditis (IE). TCD‐MES detection before clinical or radiological manifestation of stroke may serve as a criterion to allow for an earlier definite diagnosis of IE since, according to the modified Duke criteria for the diagnosis of IE, the presence of embolic events is one of the minor criteria. Moreover, documenting MES in patients with IE may also identify candidates for more aggressive therapies, such as earlier cardiac valve surgery, in order to reduce stroke risk and improve outcomes. Several studies so far have investigated the role of MES detection in patients with IE: 16%–40% of the patients with IE presented MES on TCD [22, 23]. Furthermore, Lepur and Barsic showed a significant correlation between MES and neurological deterioration in IE patients [23].

#### Small vessel disease‐associated AIS

Small vessel disease‐associated AIS is the TOAST‐subtype with the weakest association with MES detection. Only 3% of patients with small vessel disease may have MES according to the synthesis of available data [4]. This is mostly expected since lacunar strokes occurs very rarely on the grounds of embolization. Yet, a restricted number of MES may be detected, especially when the monitoring duration is prolonged, probably due to coincidental atherosclerotic disease, which can be quite common in this patient group.

#### Cryptogenic AIS

After the introduction of Embolic Stroke of Undetermined Source (ESUS) as a clinically useful construct, referring to a non‐lacunar ischemic stroke that remained cryptogenic after standard diagnostic evaluation, Higuchi et al. sought to estimate the prevalence and risk factors of MES in ESUS and highlighted the embolic nature of ESUS [24]. MES recorded unilaterally over an hour were more common in patients with ESUS (50%), followed by large artery atherosclerosis (40%), cardioembolic stroke (33%), TIA, (17%) and cerebral small vessel disease (4%), indicating the fact that the presence of MES suggests a non‐lacunar stroke. Moreover, bilateral MES detection may suggest a cardioembolic origin. In contrast, unilateral MES detection is more often associated with large artery atherosclerosis. Therefore, differentiating between unilateral and bilateral MES detection can offer insights into the probable source of emboli, aiding in etiological assessment and targeted management.

### AIS of other determined etiology

#### Cancer‐associated AIS

Numerous studies so far have demonstrated a well‐established association between cancer and stroke. Two studies have shown that MES have high prevalence in cancer‐related stroke. The first one was a single‐center study showing that 46% of patients with cancer and ischemic stroke had circulating MES on TCD as recorded over 30 min bilaterally and this proportion was even higher in those with an undetermined stroke mechanism [25]. The second study included matched patients from two centers forming three groups: the main group included patients with active solid tumor cancer and AIS and the control groups included patients with AIS without cancer or active cancer without AIS. Each group had 50 participants [26]. MES were recorded over 30 min bilaterally and detected in 32% of cancer‐plus‐stroke participants, 16% of stroke‐only participants, and 6% of cancer‐only participants (*p* = 0.005).

Furthermore, detection of cerebral MES in ESUS patients with cancer, as an indicator of arterial thromboembolism, when monitored bilaterally over 30‐min could assist not only in the diagnosis, but also in the prognosis of cancer‐related stroke, since MES were independent predictors of poor 1‐year survival [27]. Consistently, a more recent study demonstrated that among patients with AIS and active cancer, the presence of MES was associated with higher D‐dimer and C‐reactive protein levels, multiple ischemic lesions across three territories, and predicted short‐term survival [28].

#### Cancer‐associated non‐bacterial thrombotic endocarditis

**FIGURE 4:**
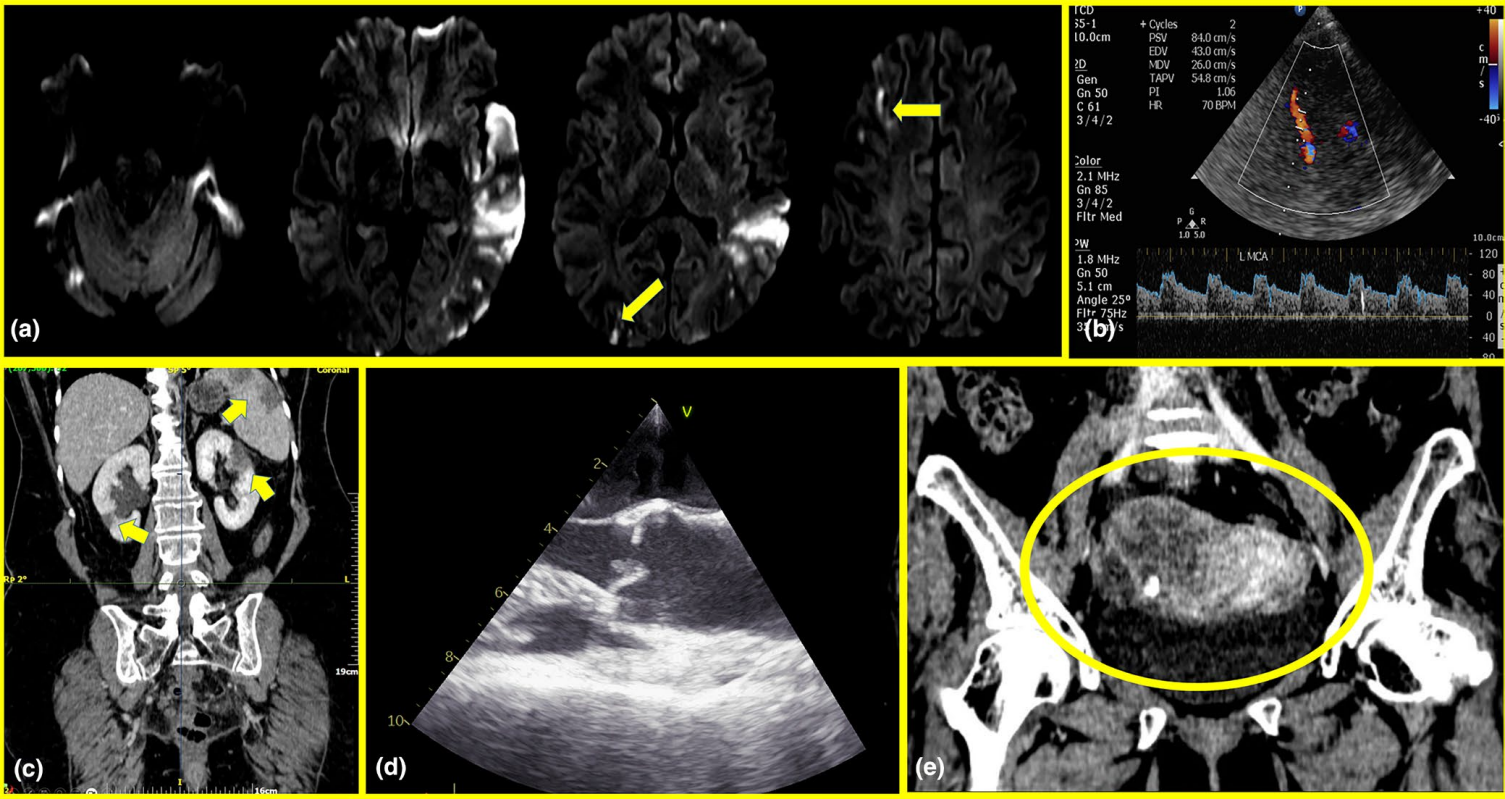
Patient with acute ischemic stroke lesions in multiple arterial territories, as shown in MRI‐Diffusion Weighted Image (a), with positive microembolic detection on the middle cerebral artery as detected by Transcranial Color‐Coded Doppler (b) and multiple visceral infarcts on CT‐scan (kidneys, spleen; (c)), due to non‐bacterial thrombotic endocarditis as detected during transesophageal echocardiogram (d), associated with ovarian mucinous cystadenocarcinoma (CT‐scan; (e)).

Non‐bacterial thrombotic endocarditis (NBTE) is a relatively rare and underdiagnosed condition that involves the formation of sterile valvular vegetations consisting of platelets and fibrin which tend to embolize. NBTE should be suspected in cancer‐associated ESUS, and an extensive diagnostic work‐up should be initiated. While the cornerstone for the diagnosis of NBTE is the transesophageal echocardiogram, MES detection in TCD is one of the core features of cancer‐associated NBTE besides high D‐dimer levels, visceral infarcts, cerebral infarcts in multiple vascular territories, as well as disseminated cancer and adenocarcinoma histology (Figure 4).

#### Cervical artery dissection

MES may also be detected in patients with carotid or vertebral artery dissection, independently of whether the dissection has been complicated with AIS or not. Among those that had indeed an AIS secondary to dissection, MES were detected in almost 50% [29].

With the aim to prevent thromboembolic complications, anticoagulation has been previously recommended for all patients with acute cervical artery dissection, unless there were contraindications, such as intracranial extension of the dissection. According to recent data, there is no difference in stroke prevention between patients treated with either antiplatelets or anticoagulants and both treatment approaches are equally recommended [30]. However, a post‐ hoc, exploratory analysis of the aforementioned study has demonstrated that anticoagulation might be preferable in patients with cervical artery dissection presenting with ischemia and no occlusion or no intracranial extension of the dissection [31]. Consequently, it may be hypothesized that, as the choice of antithrombotic treatment can be tailored in many cases, the presence of MES provides an additional decision tool in individualizing the appropriate treatment.

#### Aortic arch atheroma/dissection

Aortic arch atheroma has been associated with an almost four‐times increased stroke risk. One plausible pathophysiological scenario for this apparent association seems to be artery‐to‐artery embolism, a hypothesis further endorsed by the positive MES detection among AIS patients with aortic arch atheroma [32]. MES may also be detected in cases with ascending aortic artery dissection, with an almost 25% prevalence among those with extension to the brachiocephalic arteries [33]. Due to limited data, no definitive conclusions can be made about the clinical implications of MES identification.

#### Central nervous system (CNS) vasculitis

Stroke in younger ages coupled with multiple infarcts on imaging may be suggestive of underlying vasculitis, either primary or secondary angiitis of the CNS. The presence of MES in systemic lupus erythematosus (SLE) patients with CNS involvement has been evaluated in several studies. Dahl et al., reported that MES were detected over a monitoring time of 60 min unilaterally in 5 (9%) out of 55 SLE patients [34]. Importantly, they found a significant association between MES and cerebral infarcts. These findings were supported by a previous study demonstrating a 15% prevalence of MES in 16 SLE patients [35].

Apart from SLE‐vasculitis, embolism in terms of MES detection has been assessed in other types of vasculitis as well, such as Behçet's and Takayasu disease, indicating a potential mechanism for CNS involvement. An interesting case report described acute infarctions located predominantly in the posterior circulation with a remarkable presence of multiple MES in the posterior circulation as the initial manifestation of Takayasu arteritis [36].

#### Moyamoya disease (MMD)

MMD is characterized by chronic progressive steno‐occlusion at the terminal segment of the internal carotid artery, proximal segment of the anterior cerebral artery, and the MCA coupled with abnormal collateral networks, that often results in cerebral ischemia and hemorrhage in adults. The occurrence of MES in MMD ranges between 12.5% and 32.0% among different studies [37, 38]. An association between recent ischemic events and the presence of MES is well‐established [38]. Regarding the correlation of MES detection and the stage of the disease, there are discrepant results. Chen et al. showed no clinical association between MES detection and the stage of MMD [38]. However, Horn et al. demonstrated that MES as recorded in MCA bilaterally over 1 h were more frequent in patients with Suzuki stage II early MMD [37]. Whether patients with MMD should be treated with long‐term antiplatelet therapy as they suffer from both ischemic and hemorrhagic stroke is still under discussion. In this context, MES detection may provide an argument in favor of the initiation of antiplatelet treatment in MMD.

#### COVID‐19 associated stroke

The recent experience with COVID‐19 pandemic revealed several cerebrovascular complications associated with SARS‐CoV‐2 infection, with the risk of ischemic stroke being increased among patients infected by SARS‐CoV‐2 compared to contemporary or historical non‐infected controls. Two small studies have so far evaluated MES detection in COVID‐19 patients. The first one reports on 5 patients with confirmed COVID‐19 infection and stroke; MES were identified in three of them by TCD. Notably, MES were detected in multiple vascular territories in two of the patients with a monitoring time of 45–65 min [39]. On the contrary, in the second study, no MES were found in two COVID‐19 infected patients with confirmed large‐vessel occlusion (LVO)‐related stroke [40]. However, it should be acknowledged that the duration of TCD‐monitoring for MES detection in this study was very short (15 min).

## PROGNOSTIC SIGNIFICANCE OF MES IN AIS

Several studies aimed to determine the prognostic role of MES detection on TCD in AIS patients [41, 42]. Hao et al. sought to investigate whether there was an association between MES presence and neurological deficits among 67 AIS patients with LVO [43]. TCD was performed on Day 1 and on Day 7. Among the patients with positive MES at baseline, the disappearance of MES on Day 7 was positively correlated with neurological improvement, while patients who still had MES on Day 7 were more likely to experience neurological deterioration. Similar findings were shown in a previous study that included 114 AIS patients with MCA stenosis [14]. MES were detected in 25 (22%) patients and were more common in patients with severe stenosis (48%) compared to those with mild or moderate stenosis (15%). During the follow‐up, 12 (12%) patients had stroke recurrence in the affected MCA region, showing a significant association with the presence of MES.

Apart from intracranial stenoses, MES are also an independent risk factor for recurrent stroke and neurological deterioration in patients with extracranial carotid artery stenosis as well. When TCD was positive for MES in patients with symptomatic carotid stenosis, the early risk of recurrent stroke was approximately 8%. On the other hand, when TCD was negative for MES in the follow–up period, no ipsilateral ischemic event was recorded [8].

Regarding all stroke subtypes, in the study of Das et al. 248 TCD evaluations were performed, and stroke was detected ipsilaterally to MES detection in 97% of patients [44]. Stroke recurrence was recorded in 10.9% of patients. In a univariate analysis, the number of MES was significantly associated with recurrence. Allover, MES were considered as an independent predictor of adverse clinical outcomes, as defined by the functional disability at discharge and the increased duration of hospitalization.

Regarding stroke recurrence, several meta‐analyses have been published, consistently showing that MES detection is associated with higher odds of stroke recurrence (Table 2).

**TABLE 2:** Systematic reviews and meta‐analyses reporting on the association of microembolic signals and stroke recurrence.

Study (Year)	Patient subgroup	Number of included studies/patients	Pooled proportion of recurrent stroke (95%CI)		OR (95% CI)
			Positive MES	Negative MES	
Ritter et al. [32]	Symptomatic carotid stenosis	3/354	26% (19%–34%)	4% (91%–9%)	7.5 (3.6–15.4)
	Asymptomatic carotid stenosis	5/740	24% (8%–45%)	1% (0%–3%)	13.4 (6.5–27.4)
King et al. [41]	All AIS/TIA	8/737	19% (13%–26%)	5% (2%–9%)	3.7 (1.6–8.4)
	Symptomatic carotid stenosis	4/270	28% (20%–37%)	5% (2%–9%)	6.4 (2.9–14.0)
	Asymptomatic carotid stenosis	5/677	30% (12%–52%)	2% (0%–5%)	12.0 (2.4–59.3)
	Carotid endarterectomy	6/649	7% (4%–10%)	1% (0%–3%)	3.6 (1.4–9.2)
Tsivgoulis et al. [42]	Large artery atherosclerosis‐stroke patients with positive MES receiving antiplatelet treatment	2/205	3% (1%–6%)	NA	NA
	Large artery atherosclerosis‐stroke patients with positive MES receiving dual antiplatelet treatment	2/97	0% (0%–1%)	NA	NA
	Large artery atherosclerosis‐stroke patients with positive MES receiving single antiplatelet treatment	2/108	5% (2%–10%)	NA	Dual versus Single Antiplatelet Treatment: RD = −6% (−11% to −1%)
Sudheer et al. [4]	All AIS/TIA	19/1243	21% (12%–31%)	5% (3%–9%)	4.0 (2.4–6.8)
Sudheer et al. [29]	Cervical artery dissection	4/41	49% (27/71%)	3% (0%–14%)	11.7 (2.0–70.1)

Abbreviations: AIS, acute ischemic stroke; CI, confidence interval; MES, microembolic signals; NA, not applicable; OR, odds ratio; RD, risk difference; TIA, transient ischemic attack.

## CLINICAL SIGNIFICANCE OF MES DURING AND AFTER ACUTE REPERFUSION THERAPIES

Whether MES detection on TCD at the site of arterial occlusion could indicate clot dissolution and the beginning of recanalization of intracranial arteries in patients with AIS receiving intravenous thrombolysis was studied by Alexandrov et al. [45] Three cases were described, in which MES preceded spontaneous clinical recovery and normalization of MCA waveforms. More recently, another study reported a higher prevalence of MES detection among AIS patients that received intravenous thrombolysis compared to those that did not (29.2% vs. 18.8%, respectively), however, this difference was not statistically significant and no further conclusions were drawn [46].

Regarding endovascular treatment (EVT) and the prevalence of MES in AIS, two studies so far have been identified. The first study included 40 AIS patients who received mechanical thrombectomy due to anterior LVO [47]. TCD was performed bilaterally immediately after EVT for 60 min: MES were detected in 65% of the patients. A higher MES number was significantly associated with ipsilateral carotid occlusion or stenosis (≥50%), incomplete recanalization and inadequate collaterals. Additionally, patients with higher MES count on TCD had worse functional outcomes, higher mortality, higher distal embolization burden, and higher risk of other cardiovascular events.

The second study, with a shorter monitoring period (30 min), included 111 AIS patients with LVO of the anterior circulation who were successfully treated with mechanical thrombectomy resulting to a TICI score 2b‐3 [48]. MES were detected in 43 patients (39%). Their presence was not associated with a significant difference in the modified Rankin Scale. However, it was associated with a higher (six‐ to eight‐fold) risk of recurrent ischemic stroke or systemic embolization during the 90‐day follow‐up.

## TECHNICAL CONSIDERATIONS AND LIMITATIONS OF TCD RECORDING FOR MES DETECTION

According to the International Consensus Group on Microembolus Detection published guidelines and recommendations, studies with MES should report on (i) ultrasound device, (ii) transducer type and size, (iii) insonated artery, (iv) insonation depth, (v) algorithms for signal intensity measurement, (vi) scale settings, (vii) detection threshold, (viii) axial extension of sample volume, (ix) fast Fourier transform (FFT) size (number of points used), (x) FFT length (time), (xi) FFT overlap, (xii) transmitted ultrasound frequency, (xiii) high‐pass filter settings, and (xiv) recording time. Unfortunately, not all published studies in the field have explicitly stated those important features (Table S1).

In patients with carotid artery disease or atrial fibrillation, the frequency of MES may be relatively low; therefore, recordings of at least 1 h are recommended. However, in patients with mechanical heart valves or during the monitoring of invasive procedures, the frequency of MES is higher, allowing for a shorter recording time.

As with all TCD measurements, MES detection and reporting are dynamic and highly dependent on the investigator. Yet, multiple algorithms and setups have become available to automate MES detection. Furthermore, novel robotic TCD ultrasound systems have proven to be feasible for the evaluation of right‐to‐left shunts [49] and may also be considered in the setting of MES detection as well.

All over, there are further limitations for MES detection in AIS patients. For example, variations in the timing of MES detection can lead to inconsistent results. Early or delayed detection after stroke occurrence might yield different clinical insights, impacting the accuracy of findings. Thus, the use of different protocols for MES detection and monitoring (duration of the recording, manual vs. automated detection), introduces further variability. As shown recently, MES are more likely to be detected within the first 24 h after stroke onset and may be negative over time [50]. Additionally, since the process can be time‐consuming, the feasibility of incorporating it into routine, fast‐paced clinical workflows may be affected.

## FUTURE DIRECTIONS

Integrating MES detection into clinical decision‐making algorithms to inform treatment strategies and enhance patient outcomes in acute ischemic stroke (AIS) patients is a crucial area for future research. Additionally, repeated transcranial Doppler (TCD) monitoring using ambulatory devices to evaluate the time course of MES could serve as a valuable marker. Therefore, establishing standardized protocols for MES detection would ensure consistency across studies and clinical settings, improving the comparability and reproducibility of results.

## CONCLUSIONS

MES detection on TCD in ACI remains a useful diagnostic tool as it helps the clinicians to approach the stroke underlying mechanism by detecting and quantifying ongoing cerebral embolization and localizing an embolic source in real time. In addition, it allows monitoring and treatment individualization in stroke patients, while further determining recurrent stroke risk. MES detection has also served as a surrogate marker to evaluate secondary prevention therapies. Future clinical trials investigating both acute and secondary stroke prevention treatments will be further refined by the introduction and implementation of TCD MES detection, either for the selection of patients or as a surrogate endpoint of efficacy.

## AUTHOR CONTRIBUTIONS

**Eleni Bakola:** Writing – original draft; investigation; methodology; visualization; data curation. **Lina Palaiodimou:** Investigation; writing – original draft; methodology; visualization; data curation. **Odysseas Kargiotis:** Investigation; methodology; writing – review and editing; data curation. **Apostolos Safouris:** Investigation; methodology; writing – review and editing; data curation. **Klearchos Psychogios:** Investigation; writing – review and editing; methodology; data curation. **Theodoros Karapanayiotides:** Investigation; writing – review and editing; methodology; data curation. **Christos Moschovos:** Data curation; writing – review and editing; methodology; investigation. **Vijay K. Sharma:** Investigation; writing – review and editing; methodology; data curation. **Mark N. Rubin:** Data curation; writing – review and editing; methodology; investigation. **João Sargento Freitas:** Investigation; writing – review and editing; methodology; data curation. **Claudio Baracchini:** Data curation; writing – review and editing; methodology; investigation. **Christos Krogias:** Investigation; writing – review and editing; methodology; data curation. **Andrei V. Alexandrov:** Data curation; writing – review and editing; methodology; investigation. **Tsivgoulis Georgios:** Conceptualization; investigation; writing – original draft; methodology; validation; visualization; project administration; data curation; supervision.

## FUNDING INFORMATION

This research received no specific grant from any funding agency in the public, commercial, or not‐for‐profit sectors.

## CONFLICT OF INTEREST STATEMENT

The authors declared no conflict of interest.

## Supporting information


Table S1:

## Data Availability

Data sharing is not applicable to this article as no new data were created or analyzed in this study.
