# Supplementary material for: Microembolic signal detection in acute ischemic stroke: Clinical relevance and impact on treatment individualization—A narrative review
Source: Eur J Neurol. 2024 Dec 20;32(1):e16584. doi: 10.1111/ene.16584 (PMC11661863; doi:10.1111/ene.16584)
Supplement: Supplementary file 1 — Table S1: [file ENE-32-e16584-s001.docx]

| **Study** | **Ultrasound device** | **Transducer type and size** | **Insonated artery** | **Insonation depth** | **Algorithms for signal intensity measurement** | **Scale settings** | **Detection threshold** | **Axial extension of sample volume** | **Fast Fourier transform size** | **Fast Fourier transform length (time)** | **Fast Fourier transform overlap** | **Transmitted ultrasound frequency** | **High-pass filter settings** | **Recording time** |
| --- | --- | --- | --- | --- | --- | --- | --- | --- | --- | --- | --- | --- | --- | --- |
| **Gao et al, 2004 ^1^** | 2020 Nicolet-EME | 2 MHz probe | MCA | NR | graded color scale | NR | 5 dB | 6 mm | 128-point | NR | 61%- 71% | 2 MHz | NR | 30 minutes |
| **Horn et al., 2005 ^2^** | DWL | 2 MHz probe | MCA bilateral | NR | graded color scale | NR | 5 dB | 5mm | 128-point | NR | NR | 2MHz | 100Hz | 60 minutes |
| **Markus et al,**  **2005 ^3^** | EME Pioneer 4040; EME) | 2 MHz probe | MCA | 46 –56mm | not reported | NR | 7db | NR | 128-point | NR | >50% | 2 MHz | NR | 60 minutes |
| **Dahl et al., 2006 ^4^** | TCD2000S Nicolet | 2 MHz probe | MCA | 45–56 mm | NR | NR | NR | 10mm | NR | NR | 50-65% | 2 MHz | 100 Hz | 60 minutes |
| **Tan et al., 2008 ^5^** | TCD100M | 2 MHz probe | several arteries | 24–88 mm | power (in decibels) of the Fourier transform coefficients | NR | NR | 6mm | 128-point | 16ms | 50% | 2 MHz | 200 Hz | 30 minutes |
| **Seok et al, 2010 ^6^** | Pioneer TC 8080 | 2 MHz probe | MCA bilateral | 40-60mm | NR | NR | NR | NR | NR | NR | NR | 2 MHz | NR | 30 minutes |
| **Chen et al, 2018 ^7^** | EMETC8080 | 2 MHz probe | MCA | NR | NR | NR | NR | NR | NR | NR | NR | 2 MHz | NR | 30 minutes |
| **Ha et al., 2019 ^8^** | Pioneer TC 8080 | 3 MHz probe | MCA bilateral, basilar | 40–60 mm/ 80-100mm | NR | NR | NR | NR | NR | NR | NR | 2 MHz | NR | 30 minutes |
| **Higuchi et al, 2020 ^9^** | SONARA | 2 MHz probe | MCA | 45-65 mm | NR | NR | NR | NR | NR | NR | NR | 2 MHz | NR | 60 minutes |
| **Batra et al., 2021 ^10^** | DWL Doppler-BoxX | 2 MHz probe | several arteries | NR | NR | NR | NR | 8mm | NR | NR | NR | 2 MHz | NR | 45-65 minutes |
| **Navi et al., 2021 ^11^** | Spencer ST3 | 2 MHz probe | MCA bilateral | 45–65mm | NR | NR | NR | NR | NR | NR | NR | 2 MHz | NR | 30 minutes |
| **Toi et al, 2024 ^12^** | SONARA | 2 MHz probe | MCA | 45–65mm | NR | NR | NR | NR | NR | NR | NR | 2 MHz | NR | 60 minutes |

NR: not reported

**References used in the Supplement**

1. Gao S, Wong KS, Hansberg T, Lam WW, Droste DW, Ringelstein EB. Microembolic signal predicts recurrent cerebral ischemic events in acute stroke patients with middle cerebral artery stenosis. Stroke. 2004;35:2832-6.

2. Horn P, Lanczik O, Vajkoczy P, Daffertshofer M, Bueltmann E, Werner A, et al. Hemodynamic reserve and high-intensity transient signals in moyamoya disease. Cerebrovasc Dis. 2005;19:141-6.

3. Markus HS, MacKinnon A. Asymptomatic embolization detected by Doppler ultrasound predicts stroke risk in symptomatic carotid artery stenosis. Stroke. 2005;36:971-5.

4. Dahl A, Omdal R, Waterloo K, Joakimsen O, Jacobsen EA, Koldingsnes W, et al. Detection of cerebral embolic signals in patients with systemic lupus erythematosus. J Neurol Neurosurg Psychiatry. 2006;77:774-9.

5. Tan TY, Chen TY. Detection of microembolic signals using power M-mode Doppler sonography in acute stroke patients with intracranial artery stenosis. J Clin Ultrasound. 2008;36:422-6.

6. Seok JM, Kim SG, Kim JW, Chung CS, Kim GM, Lee KH, et al. Coagulopathy and embolic signal in cancer patients with ischemic stroke. Ann Neurol. 2010;68:213-9.

7. Chen X, Liu K, Wu X, Wang S, Li T, Xing Y. Microembolic Signals Predict Recurrence of Ischemic Events in Symptomatic Patients with Middle Cerebral Artery Stenosis. Ultrasound Med Biol. 2018;44:747-55.

8. Ha J, Lee MJ, Kim SJ, Park BY, Park H, Cho S, et al. Prevalence and Impact of Venous and Arterial Thromboembolism in Patients With Embolic Stroke of Undetermined Source With or Without Active Cancer. J Am Heart Assoc. 2019;8:e013215.

9. Higuchi E, Toi S, Shirai Y, Hoshino T, Ishizuka K, Shimizu S, et al. Prevalence of Microembolic Signals in Embolic Stroke of Undetermined Source and Other Subtypes of Ischemic Stroke. Stroke. 2020;51:655-8.

10. Batra A, Clark JR, LaHaye K, Shlobin NA, Hoffman SC, Orban ZS, et al. Transcranial Doppler Ultrasound Evidence of Active Cerebral Embolization in COVID-19. J Stroke Cerebrovasc Dis. 2021;30:105542.

11. Navi BB, Sherman CP, Genova R, Mathias R, Lansdale KN, LeMoss NM, et al. Mechanisms of Ischemic Stroke in Patients with Cancer: A Prospective Study. Ann Neurol. 2021;90:159-69.

12. Toi S, Higuchi E, Hosoya M, Arai S, Ishizuka K, Mizuno T, et al. Association of Transcranial Doppler Microembolic Signal With Short-Term Mortality in Acute Ischemic Stroke and Active Cancer. J Am Heart Assoc. 2024;13:e033634.
